# Optimization of mosquito egg production under mass rearing setting: effects of cage volume, blood meal source and adult population density for the malaria vector, *Anopheles arabiensis*

**DOI:** 10.1186/s12936-017-1685-3

**Published:** 2017-01-24

**Authors:** Wadaka Mamai, Nanwintoum S. Bimbile-Somda, Hamidou Maiga, José Guillermo Juarez, Zaynab A. I. Muosa, Adel Barakat Ali, Rosemary Susan Lees, Jeremie R. L. Gilles

**Affiliations:** 1 Insect Pest Control Laboratory, Joint FAO/IAEA Division of Nuclear Techniques in Food and Agriculture, Vienna, Austria; 2 Center for Health Studies, Universidad del Valle de Guatemala, Guatemala, Guatemala; 3 Tropical Medicine Research Institute, National Centre for Research, PO Box 1304, Khartoum, Sudan; 4 LITE (Liverpool Insect Testing Establishment), Vector Biology Department, Liverpool School of Tropical Medicine, Pembroke Place, Liverpool, L3 5QA UK

**Keywords:** Sterile insect technique, Mass production cage, *Anopheles arabiensis*, Egg production

## Abstract

**Background:**

*Anopheles arabiensis* is one of the major malaria vectors that put millions of people in endemic countries at risk. Mass-rearing of this mosquito is crucial for strategies that use sterile insect technique to suppress vector populations. The sterile insect technique (SIT) package for this mosquito species is being developed by the Insect Pest Control Subprogramme of the Joint FAO/IAEA Division of Nuclear Techniques in Food and Agriculture. To improve mass-rearing outcomes for *An. arabiensis,* the question of whether the egg production by females would be affected by the size of the adult holding cages, the source of the blood meal and the total number of pupae that could be loaded into the cages was addressed and finally the impact of adding additional pupae to the cage daily to maintain adult numbers on egg productivity assessed.

**Methods:**

Mass production cages of two different volumes, two different sources of blood meal (bovine and porcine) and two different population densities (cages originally loaded with either 15,000 or 20,000 pupae) were tested and evaluated on the basis of eggs produced/cage or per female. Males and females pupae with a ratio of 1:1 were added to the cages at day 1 and 2 of pupation. The emerging adults had constant access to 5% sugar solution and blood fed via the Hemotek membrane feeding system. Eggs were collected either twice a week or daily. A generalized linear model was used to identify factors which gave significantly higher egg production.

**Results:**

Neither cage volume nor blood meal source affected egg production per cage or per female. However, increasing population density to 20,000 pupae had a negative effect on eggs produced per cage and per female. Although high density negatively impacted egg production, adding 1000 daily additional pupae compensating for daily mortality resulted in a substantial increase in egg production. Moreover, in all tests the first and the third egg batches collected were significantly higher than others eggs batches. With the equipment and protocols described here and routinely used at the Insect Pest Control Laboratory (IPCL), it was possible to produce up to 120,000 eggs/cage/day.

**Conclusion:**

These results demonstrated that 15,000 is the optimal number of pupae to be loaded into the *Anopheles* Mass production cages. Under this condition, an average of 40 eggs per female was obtained for five gonotrophic cycles. However, an improvement in egg production can be achieved by daily addition, to the original 15,000 pupae, of one thousand pupae a day. Interestingly, feeding females with bovine or porcine blood using both large and small versions of the mass production cage did not affect egg productivity.

## Background

The urgent need to better control mosquito numbers and interrupt disease transmission has guided much mosquito research in laboratories worldwide. Such research including the study of the biology, physiology, anatomy, genetics, taxonomy and ecology usually use individual or quantity rearings of mosquitoes. The goal of insect rearing is to provide reliable, affordable sources of high-quality insects. For most of these purposes or for the routine colony maintenance, *Anopheles* mosquitoes were reared in standard laboratory rearing trays containing water that held less than 500 larvae [1, 2]. Pupae, individually picked, were transferred into a small bowl that is placed inside a small rearing cage (30 × 30 × 30 cm) for emergence and maintenance. However, rearing for large-scale needs requires a variety of improvement in methodology and equipment. The use of the sterile insect technique (SIT) for the control of pest insects as part of an integrated, area-wide approach is widely accepted. This technique utilizes radiation-sterilized individuals, which are released into the field and the wild population of the pest is then suppressed by the occurrence of sterile mating [3–5].

In order to reach a sustainable field population reduction, one of the key challenges when applying SIT is the production of sufficient mosquitoes to achieve the target production level of males to be released and for colony replacement [4, 6]. For this reason, it is necessary to continually produce large numbers of eggs (millions of eggs/day) to fill several tray-rack larval rearing units [7, 8] in order to reach a daily operational level able to sustain continuous large scale operation activities. With revived interest in recent years for the use of sterile male release for mosquito control [9–15], there is a requirement for the development of more efficient and economical methods to produce large numbers of sterile male mosquitoes. Therefore, in the production facility, pressure is directed towards parameters which are important for high egg productivity.

Since 2005, the Insect Pest Control Laboratory (IPCL) of the joint Food and Agriculture Organization/International Atomic Energy Agency (FAO/IAEA) division of Nuclear Techniques in Food and Agriculture has developed dedicated technology and procedures [16] to support mosquito vector control programmes in Member States that use the SIT as a component of area-wide integrated pest management (AW-IPM). The mass rearing of mosquitoes is a key element of the application of the SIT, and so custom equipment has been designed for both larval and adult components of *Anopheles arabiensis* rearing [7, 17, 18]. A prototype of mass production cage (MPC) has been developed [18], and designed to minimize handling and the opening of the cages during operations such as blood feeding and egg collection. This cage exists in two different sizes, a large cage with dimensions length 200 cm × width 20 cm × height 100 cm (400-litre volume) and a small cage length 200 cm × width 10 cm × height 100 cm (200-litre volume). Both cages include: (i) an external blood feeding system (modified Hemotek system, Discovery Workshops, Lancashire, UK), (ii) a built-in sugar feeding system, and (iii) an oviposition system located at the bottom of the cage. Although the cages have been transferred to Sudan and South Africa for validation under local conditions, and improvements in equipment and techniques have been made as a result, there is still a need to fully evaluate the productivity of such equipment and to quantify how operational parameters affect egg productivity.

The objective of this study was to optimize the rearing method to ensure high egg productivity of *An. arabiensis* in the mass rearing cage prototypes (*Anopheles* MPC, large and small). Experiments were conducted to determine the impact of four parameters on eggs production: (i) cage volume, (ii) blood meal source, (iii) total number of pupae introduced into the cages, and (iv) loading cages with all pupae on cage set-up compared to cages topped up with a daily addition of further pupae. A greater understanding of the effects of these factors would allow to define the conditions under which *An. arabiensis* adults should be maintained in order to maximize the effectiveness of the rearing process.

## Methods

### Mosquito colony

Experiments were conducted using an *An. arabiensis* Dongola strain originating from the Northern State of Sudan (Tropical Medicine Research Institute, Khartoum). This colony has been maintained at the IPCL since 2005 under controlled temperature, humidity and lighting conditions (27 ± 1 °C, 70 ± 10% relative humidity, 12:12 h light:dark, including 1 h dusk and 1 h dawn). This colony has no known insecticide resistance.

Pupae used for this study were reared following the *An. arabiensis* mass-rearing procedure developed at the IPCL [7, 16, 19, 20]. Eggs were hatched and larvae reared to pupation in the larval mass rearing rack developed by Balestrino et al. [7, 8]. Each tray was filled with 4 L of de-ionized water the day before adding the eggs to allow the water to reach room temperature. Using the egg quantification method developed by Maiga et al. [20], 50 aliquots of 4000 eggs were added to each tray in a plastic ring floating on the surface of the water. Larvae were fed with the IAEA liquid diet (5 g/L tuna meal, 5 g/L bovine liver powder and 4.6 g/L vitamin mix) following the published protocol [19], and 24 h after the first pupae were observed the rack was tilted to collect larvae and pupae. Pupae were separated from larvae by swirling in an Erlenmeyer flask with tap water [17, 21] and retained for use in all experiments.

### Mass production cage: description and general rearing protocol

**Fig. 1:**
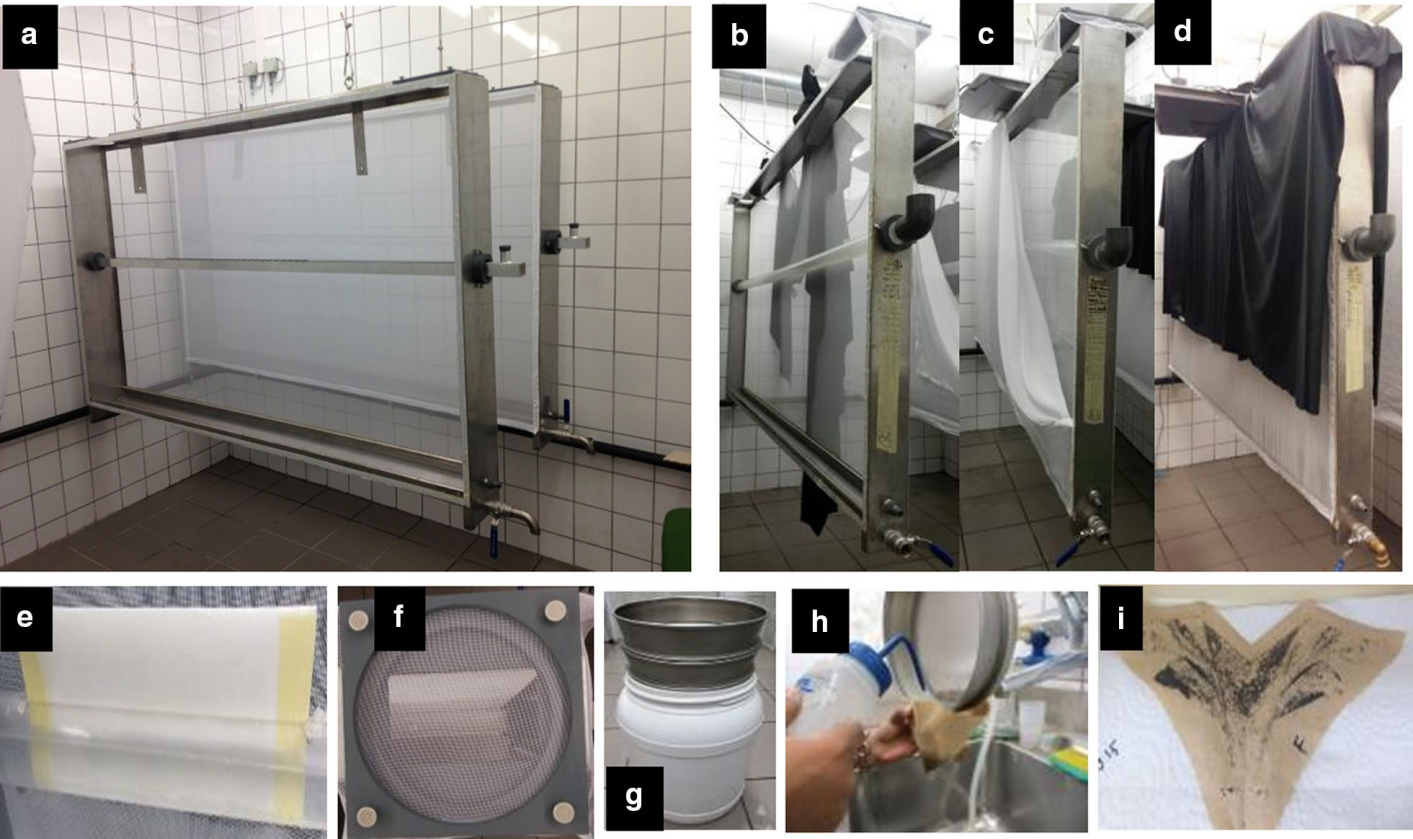
Experimental set up of the *Anopheles* MPC. View of the device described in Balestrino et al. [7] (**a**); MPC without the net prior to adding pupae (**b**); MPC with the net partially attached after loading the pupae (**c**); MPC fully assembled with the net and black lining (**d**); sugar feeding device with filter paper (**e**); the blood feeding port (**f**); collection of eggs (**g**–**i**)

The *Anopheles* mass production cage (MPC) used in these experiments was previously described by Balestrino et al. [18] and improved by Maiga et al. (pers. comm.) as follow: the aluminium sugar feeder previously described [18] has been replaced with a plastic cylindrical tube (2.2 m long and 50 mm in diameter), and sugar solution is provided to adults mosquitoes from the sugar feeder using a filter paper (Whatman paper, 2589 A Bogen sheets, 580 × 580 mm). The cages were hung from the ceiling in the colony room described above and were three-quarters covered with a black cloth to create an artificial horizon designed to stimulate natural mating behaviour. The experimental set up of the MPC is presented in Fig. 1. Pupae used to load cages were volumetrically quantified using a modified 50-mL conical centrifuge tube and distributed into lots of approximately 2500 pupae in square plastic cups (10 by 10 by 6 cm) containing 200 mL of dechlorinated water. Cups containing pupae were then placed in the *Anopheles* MPC at specific densities and allowed to emerge. From each cohort of pupae, one-hundred pupae were placed in a cage (30 × 30 × 30 cm, BugDorm-1H; MegaView, Taichung, Taiwan) to determine the adult sex ratios of the cage populations. The emerging adults in the *Anopheles* MPC were given ad libitum access to 5% sugar solution from the sugar feeder using a filter paper (Whatman paper, 2589 A Bogen sheets, 580 × 580 mm). After emergence was complete, cups were removed and dead pupae were counted. Dead adults were also removed by draining water from the cage. Male and female mortality was recorded to evaluate adult survival and to determine the number of females remaining in the cage, in order to estimate the number of eggs laid per female in each batch.

**Fig. 2:**
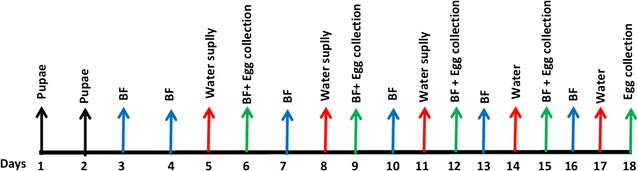
Design of the experimental rearing procedure for adult *Anopheles arabiensis.* (*BF*=blood feeding)

Females were offered a first blood meal on the 3rd day after loading of cages (Fig. 2). Blood feeding was done in the mornings (between 9 and 11am) artificially using the Hemotek membrane feeding system (Discovery Workshops, Lancashire, UK) which maintains the blood at 37  ±  2 °C [16, 22]. After two blood feedings, water was then supplied for oviposition the following day, in the trough at the bottom of the cage. The hosepipe is used to fill the trough with sufficient dechlorinated water (but without overfilling otherwise eggs get stuck under the internal pipe). The following day eggs were collected, rinsed and placed on a piece of sterile filter paper, and allowed to air dry for 4 h [23] and then quantified using the method developed by Maiga et al. [20].

### Experiment 1: assessing effect of adult population density and cage volume on survival and egg productivity

The number of adult mosquitoes per square centimetre of vertical resting surface in a cage (so-called Density-Resting Surface, or DRS) is considered an important parameter connected to mating, feeding and longevity [18]. Preliminary experiments using an initial loading of 10,000 pupae resulted in a relatively low egg production (unpublished data). Therefore, in these trials 15,000 or 20,000 pupae were used corresponding to a density resting surface of ca. 2.9 and 2.2 mosquitoes per cm^2^ respectively (for the large cage size) or 2.8 and 2.1 cm^2^ respectively (for the small cage size). Two *Anopheles* MPC of different volumes available at the IPCL were used to test the effect of adult density on egg production: a ‘large MPC cage’ with dimensions 200 L by 20 w by 100 cm h (400 litre volume) and presenting a vertical resting surface of 44,000 cm^2^ and a ‘small MPC cage’ 200 L by 10 w by 100 cm h (200 litre volume) with a vertical resting surface of 42,000 cm^2^. Mosquitoes had constant access to a 5% sugar solution and females were blood fed for 2 h with 50 mL of defrosted bovine blood via a Hemotek membrane feeding system as described above. Eggs were collected at 72 h intervals starting from the day after the first blood meal for a period of 15 days (see Fig. 2). For each density and each cage volume, three replicates were done.

### Experiment 2: assessing effect of blood meal source on egg productivity

Two sources of blood, bovine and porcine were compared. Commercially available, quality-tested fresh defibrinated bovine blood, which had been frozen for storage [24], and porcine blood from a local abattoir and used routinely for *Aedes* mosquito colony maintenance at IPCL. Pupae from a single cohort were divided into lots of 15,000 pupae and one lot loaded into each MPC cage. Similar protocols for blood feeding, sugar feeding and eggs collection were used as described above and in Fig. 2, with females blood fed with either defrosted bovine blood or fresh porcine blood. Three replicates were performed per MPC cage size and per blood type.

### Experiment 3: impact of daily addition of pupae into the *Anopheles* MPC on egg productivity

This experiment was set up to evaluate whether adding a further 1000 pupae daily in addition to the original pupae loaded into the cage on setup, to compensate for adult mortality, could improve egg production. Large MPC cages were used in this experiment. Control cages were loaded only with an initial 15,000 pupae and the experimental cages were initially loaded with 15,000 and then approximately 1000 pupae were added daily for 24 days, except for 2 days (day 5 and 6) when pupae were not available. Each day for 25 days starting four days after adult emergence, females were blood fed with defrosted bovine blood, eggs collected and number estimated, and dead bodies counted and removed. All other conditions were as described above.

### Statistical analysis

The number of eggs laid per female in each batch was determined by dividing the total number of eggs produced by the number of surviving females present before we supplied water for egg laying. Data were analysed using SPSS V13 (IBM, USA) and Excel (Microsoft, USA). A generalized linear model (GLM) multivariate analysis with an identity link function [25, 26] was used to analyse factors affecting *An. arabiensis* females’ total egg production (fecundity). Two models were tested to determine the best fit for our data, for this the akaike corrected information criterion was used to select the appropriate model. The model used was based on fixed factor main effects for “egg batch”, “total pupae”, and “cage size”, a two-way interaction between “cage size*total pupae”, and a random factor for “number of females”. Adult survival curves were estimated using the Kaplan–Meier method. The difference between treatments was compared using the log-rank test. For experiment 3, a paired *t* test was conducted using GraphPad prism software to examine the effect of daily egg production between the control group (15,000 pupae) and the intervention (15,000 plus 1000 additional pupae daily). Moreover, a one-way ANOVA was implemented to determine the days on which the addition of 1000 pupae had the greatest effect on egg production.

## Results

### Experiment 1: effect of adult density and cage volume on egg production and adult survival

**Fig. 3:**
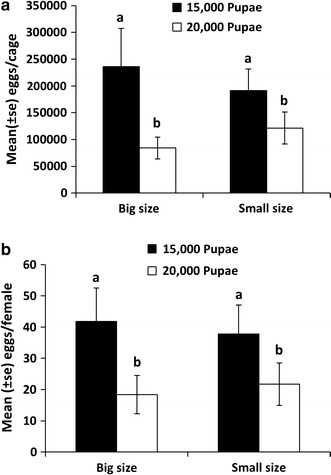
Mean number of eggs laid by *Anopheles arabiensis* per cage (**a**) and per female (**b**) as a function of cage size and number of adult per cage *Different letters* indicate statistically different results between treatments

Adult density had a significant effect on egg production (GLM, df = 1, F = 6.85, P = 0.012). Indeed, the average total number of eggs produced per cage decreased significantly in cages with high population density (Fig. 3a, GLM, df = 1, F = 6.85, P = 0.012). Fecundity, expressed as the number of eggs laid per female, also differed according to the adult density (Fig. 3b, GLM, df = 1, F = 7.320, P = 0.010). The mean number of eggs collected was 42,708 ± 11,317 and 20,527 ± 6660 per batch for the 15,000 and 20,000 pupae treatments, respectively.

The volume of the cage had no effect on the total number of eggs laid per cage (Fig. 3a, GLM, df = 1, F = 0.008, P = 0.931), or on fecundity (Fig. 3b, GLM, df = 1, F = 0.002, P = 0.965). The interaction between adult density and cage size was not significant for total egg production or fecundity (GLM, df = 1, F = 0.931, P = 0.340 and GLM, df = 1, F = 0.252, P = 0.619), respectively.

**Fig. 4:**
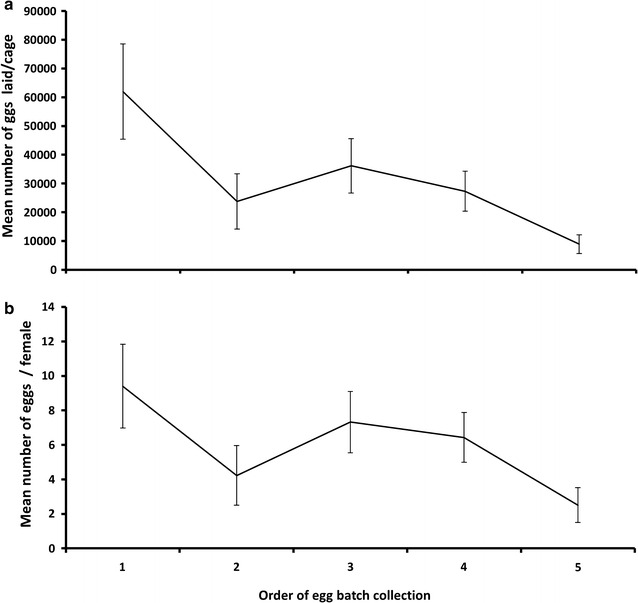
Mean number (mean ± SE) of eggs laid per cage (**a**) and per female (**b**) as a function of order of egg batch collection in *Anopheles arabiensis*

Average egg production and fecundity varied between egg batches regardless of adult density or cage size (Fig. 4, GLM, df = 4, F = 4.287, P = 0.006 and GLM, df = 4, F = 2.710, P = 0.043 for total eggs and fecundity, respectively). Total egg lay and fecundity were significantly greater at the first collection compared to the second and fifth egg collections (P = 0.003 and P = 0.038 for total egg lay and fecundity, respectively). The number of eggs in the first and the third egg collections were 6.97 and 4.07 times greater, respectively, than the fifth egg collection. The maximum egg production occurred between the first and the third egg collections (approximately 75% of the productive capacity). With 15,000 pupae loaded in the cages, an average of 39.76 ± 10.04 (mean ± SE) eggs was laid by each female throughout five gonotrophic cycles.

**Fig. 5:**
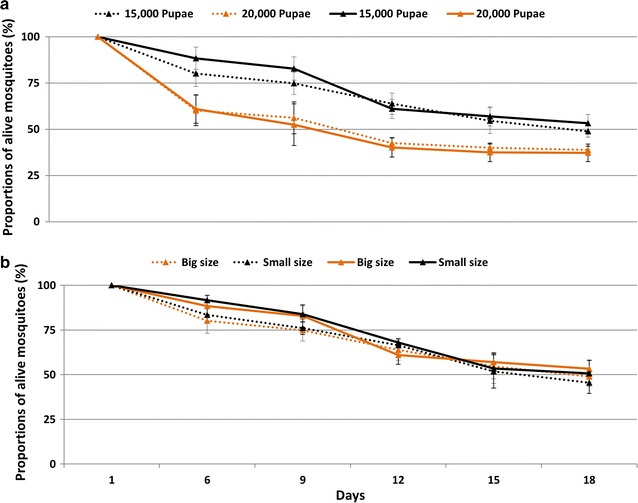
Survival of male (*dotted lines*) and female (*solid lines*) *Anopheles arabiensis* as a function of number of adult per cage (**a**) and cage size (**b**)

Mortality rate was significantly greater in females (Fig. 5a, Log-rank (Mantel-Cox) test, χ^*2*^ = 6.539, df = 1, P = 0.0106) and in males (Log-rank (Mantel-Cox) test, χ^*2*^ = 6.148, df = 1, P = 0.0132) when cages were loaded with 20,000 pupae than with 15,000 pupae, but no statistical difference was found in mortality rates in males or females between cage types (graphical observation, Fig. 5b; Log-rank (Mantel-Cox) test, χ^*2*^ = 0.3749, df = 1, P = 0.5403 and Log-rank (Mantel-Cox) test for females, χ^*2*^ = 0.1510, df = 1, P = 0.6976 for males).

### Experiment 2: effect of blood meal source

Whether mosquitoes were fed with bovine or porcine blood had no significant effect on egg production per cage (GLM, df = 1; F = 0.274, P = 0.78), or fecundity (GLM, df = 1, F = 1.444, P = 0.256). Moreover, the mortality rate was not significantly different between blood meal treatments in males (Log-rank (Mantel-Cox) test, χ^*2*^ = 1.102, df = 1, P = 0.2939) or females (Log-rank (Mantel-Cox) test, χ^*2*^ = 0.0017, df = 1, P = 0.9664).

### Experiment 3: impact of daily addition of pupae into the mass rearing cages on eggs productivity

**Fig. 6:**
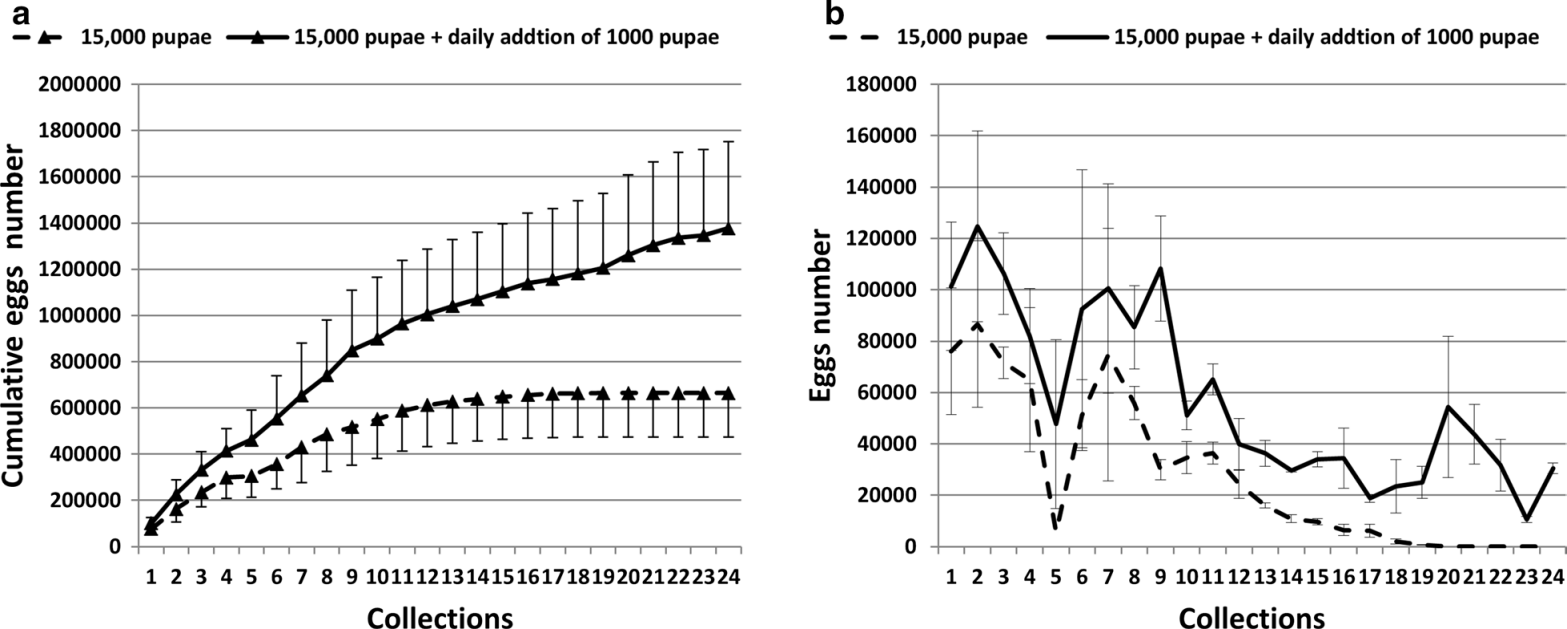
Cumulative egg number (mean ± SE) (**a**) and daily mean (± SE) number of eggs (**b**) laid by *Anopheles arabiensis* adults reared in mass production cages, comparing cages only loaded with an initial fixed number of pupae (*dotted line*) with those to which 1000 pupae were also added daily (*solid line*)

The impact of 1000 additional pupae daily in the cage containing 15,000 pupae on egg production is presented in Fig. 6. There were a significant increase in the cumulative eggs laid/cage in the treatment compared to control (Fig. 6a, Paired *t* test, t = 9.115, df = 23, P < 0.0001). A significant increase in the experimental cage was also observed when comparing the mean number of eggs laid daily (Fig. 5b, Paired *t* test, t = 8.425 df = 18, P < 0.0001). The variation in the number of eggs collected daily from the control and experimental cages followed a similar trend, with no significant difference in size of egg batch from the first to the seventh egg collection, but significant increase in the experimental cage appearing from the eighth egg collection onwards. The average total number of eggs laid per cage over 24 days was 660,325 ± 137,843 and 1,354,400 ± 164,680 for control and experimental cages, respectively. The number of eggs laid in the experimental cage was 2.05 times greater than in the control. However, over a period of the first 15 days, when a total of 30,000 pupae had been added to the intervention cage, making it equivalent to two control cages of adults, the intervention cage produced 1,190,516 ± 137,842 eggs per cage compared with 660,325 ± 137,843 eggs in the control cage. The intervention cage produced 1.8 times more eggs than the control cage over this period. 50% mortality in males and females occurred within the first 10 days post-emergence. By 18 days post-emergence, total mortality was 56%.

## Discussion

Pilot studies prior to SIT application against *An. arabiensis* mosquitoes are currently being undertaken in two endemic countries, Sudan and South Africa [4, 12, 27]. Maximizing egg production is important for the mass-rearing of any insect species, and the optimization work described here provides important insights into the development of effective standard operating procedures (SOPs) for producing large numbers of eggs in a mass-rearing setting. A number of factors were evaluated: blood meal source, total number of pupae introduced into the cages, cage loading strategy and cage volume.

To produce offspring, a female must take a blood meal, develop eggs, and lay her eggs at a suitable oviposition site, and the blood meal provides essential nutrients for eggs production and reproductive fitness. Past research has shown a variation in the feeding preferences between *Anopheline* species, ranging from those that feed on a wide range of mammals and birds, to those that feed on just one species [28–30]. Several haematological properties, including biochemical composition and red cell density, vary between vertebrate species [31, 32] and could influence its nutritional value and subsequent reproductive fitness of mosquitoes that imbibe it [28, 32]. When rearing mosquito vectors in the laboratory, it is important that a blood source is selected that will facilitate predictably high egg production, both for routine colony maintenance and for experimentation, and finding the best blood source is challenging [33]. Results showed that whether a blood meal from a pig or from a cow was used there was no effect on egg productivity in colonized *An. arabiensis* under conditions of mass-rearing. It is important to note that the bovine blood tested had first been frozen and defrosted for use, while the porcine blood was always used fresh or after refrigerated storage. This is particularly important in the context of mass-rearing as fresh blood may not always be available, for example from the local slaughterhouse, on the day it is needed; it has been shown that blood can be stored under refrigeration for weeks or for several months if frozen, and still facilitate good levels of egg production. *Aedes* spp. have been shown to feed poorly or not at all on blood that had been previously frozen [34], but *An. arabiensis* is known for its more zoophilic proclivities [35, 36] and often show plastic responses in host feeding patterns, readily diverting to feeding on the most common or most amenable host(s). The explanation for the lack of influence of blood origin may alternatively be either these two blood meals are similar in quality in terms of amino acid composition in supporting egg development and ultimately egg production, or that blood source and quality are irrelevant for reproductive fitness in *An. arabiensis* which expresses only weak host preference. In contrast, in the tsetse fly, *Glossina morsitans*, blood source has a strong impact on fecundity [37], with those feeding on pig blood producing more offspring than those feeding on cow blood. However, remarkably, no relationship between the preferred host and optimum reproductive output is reported. In the context of the development of a system for mass-rearing insects, where efficiency, economics and availability of blood source are of the utmost importance [38], this result is interesting, as bovine blood and porcine blood could be used without any effect on egg production.

As insect rearing conditions become more crowded, their survival and fecundity usually decrease [39, 40], and indeed in this study high adult density adversely affected egg production in *An. arabiensis.* The decline in egg production with density might be related to competition among females for blood. Although demonstrating intraspecific competition in mass-rearing cages in hematophagous insects is extremely difficult, physical access to blood feeders was probably limited due to increased stocking density, based on authors’ observations. Kelly et al. [41] demonstrated that increasing rearing densities of female sandflies are associated with smaller blood meals in female *Lutzomyia longipalpis*. It is also likely that high density conditions constitute a form of stress which could negatively affect the performance and reproduction of the adults. Reproductive output has been shown to decline with increasing population density in many populations, as a result of direct competition for limited resources, elevated stress levels from intraspecific interactions [42–46]. In the early experiments with 10,000 pupae added per cage, egg production was lower than when 15,000 pupae were added, but later experiments found that 30,000 pupae/cage resulted in a dramatically reduced egg production. Thus, density is clearly an important parameter and must be optimized to maximize egg production.

In these experiments, neither egg production/cage nor female fecundity were affected by the volume of the cage. It was expected that increasing the volume would provide more space for mating and would result in increased inseminated females. Although the original hypothesis was not supported by the data, this observation does not mean that in all cases the volume of the cage cannot affect egg productivity. It may depend on the value of the resting surface, as a 1.8 density-resting surface value is generally reported to promote suitable adult mosquito-rearing conditions [1]. In the conditions of the present study, the resting surface area in both cages were almost the same and above the value of 2. In the Mediterranean fruit fly (Diptera: Tephritidae) and *Anastrepha oblique* (Diptera:Tephritidae), Liedo et al. [47] and Orozco-Davila et al. [48], respectively, demonstrated that an increase in the surface resting area within adult cages of the mother colony, as well as the use of low adult cage density during rearing resulted in strains with higher mating competitiveness. Thus, a role of the cage volume cannot be conclusively ruled out, and specifically internal surface area, on egg productivity. Further studies will be carried out to determine the effect of different resting surface areas on egg productivity. However, the MPC should be of adequate size for easy handling and mating.

One purpose of the present study was to look for a suitable rearing method from the standpoint of developing space-efficient cages with potential for saving time and reducing associated costs of mass-rearing of *An. arabiensis.* Cages loaded with an initial 15,000 pupae with 1000 additional pupae added daily gave acceptable results in term of egg production and offer an advantage over cages with only an initial load of 15,000 pupae. In the latter case twice as many cages would need to be maintained to produce the same number of eggs, considerably increasing the rearing costs, and making blood feeding, for example, more time consuming. For mass rearing purposes, this is desirable and necessary in order to produce large numbers of insects in an efficient manner, keeping costs below a threshold acceptable for an operational SIT programme.

## Conclusion

Efficient rearing methods and cost effective equipment which maximize egg production are essential to reliably produce the large quantity of sterile males for timely releases required for a successful area-wide integrated vector control programme with an SIT component. This study, demonstrated that it is possible to attain sufficiently high egg production from *An. arabiensis* mosquitoes for a release programme using currently available *Anopheles* mass rearing cages (MPC), both large and small versions, when feeding females with either bovine or porcine blood. The number of pupae initially loaded into the MPC could not exceed a threshold of 15,000 before egg productivity reduced significantly. However, the addition of further pupae daily to an MPC can help to increase egg production while reducing production costs, space and time handling. Results from this study should be incorporated into existing mass-rearing guidelines [23] and taken into consideration when mass-rearing *An. arabiensis* mosquitoes for SIT programmes, and others relying on large scale production of mosquitoes, to make the most efficient use of available resources and effectively manage adult rearing cages to meet high productivity goals.

## Abbreviations

SIT: sterile insect technique
RH: relative humidity
IPCL: Insect Pest Control Laboratory
FAO: Food and Agricultural Organization
IAEA: International Atomic Energy Agency
AW-IPM: area-wide integrated pest management
MPC: mass production cage
LD: light: dark
SOP: standard operating procedures
